# Peripheral and Autonomic Neuropathy in South Asians and White Caucasians with Type 2 Diabetes Mellitus: Possible Explanations for Epidemiological Differences

**DOI:** 10.1155/2017/1273789

**Published:** 2017-03-20

**Authors:** Abd A. Tahrani, Q. A. Altaf, Milan K. Piya, Anthony H. Barnett

**Affiliations:** ^1^Institute of Metabolism and Systems Research, University of Birmingham, Birmingham, UK; ^2^Department of Diabetes and Endocrinology, Heart of England NHS Foundation Trust, Birmingham, UK; ^3^Centre of Endocrinology, Diabetes and Metabolism, Birmingham Health Partners, Birmingham, UK; ^4^Department of Diabetes and Endocrinology, Derby Teaching Hospitals NHS Foundation Trust, Derby, UK

## Abstract

*Objectives.* To compare the prevalence of diabetic peripheral neuropathy (DPN) and that of cardiac autonomic neuropathy (CAN) between South Asians and White Caucasians with type 2 diabetes and to explore reasons for observed differences.* Methods.* A cross-sectional study of casually selected South Asian and White Caucasian adults attending a hospital-based diabetes clinic in the UK. DPN and CAN were assessed using the Michigan Neuropathy Screening Instrument (MNSI) and heart rate variability testing, respectively.* Results.* Patients (*n* = 266) were recruited (47.4% South Asians). DPN was more common in White Caucasians compared to South Asians (54.3% versus 38.1%, *p* = 0.008). Foot insensitivity as assessed by 10 g monofilament perception was more common in White Caucasians (43.9% versus 23.8%, *p* = 0.001). After adjustment for confounders, White Caucasians remained twice as likely to have DPN as South Asians, but the impact of ethnicity became nonsignificant after adjusting for adiposity measures or height. No difference in prevalence of standardized CAN test abnormalities was detected between ethnicities. Skin microvascular assessment demonstrated that South Asians had reduced heating flux but preserved acetylcholine response.* Conclusions.* South Asians with type 2 diabetes have fewer clinical signs of DPN compared to White Caucasians. Differences in adiposity (and its distribution) and height appear to explain these differences.

## 1. Introduction

Diabetic neuropathy (DN) is the most common form of neuropathy in the Western world and one of the most troublesome complications of diabetes mellitus, resulting in great morbidity (e.g., it is the leading cause of nontraumatic lower limb amputation), mortality, and significant economic burden [1–3]. The reported prevalence of diabetic peripheral neuropathy (DPN) and diabetic autonomic neuropathy (DAN) varies significantly depending on the population studied and the methods used [2–9]. Many factors have been implicated in the pathogenesis of DN including nonmetabolic factors such as age and height [3, 6, 10, 11], as well as metabolic factors such as hyperglycemia, hypertension, dyslipidemia, waist circumference, and obesity [3, 12–14]. These metabolic factors stimulate multiple pathways resulting in direct cellular damage and functional and/or structural defects involving the extracellular matrix and/or microvasculature [15, 16]. Disease-modifying treatments are still lacking (with the exception of improved glycaemic control) [12, 15]. Hence, improved understanding of DN pathogenesis is important in order to identify new treatment targets [15].

South Asians with type 2 diabetes seem to be at lower risk of developing DPN resulting in a lower incidence of foot ulcerations and amputations [17–19]. Such a difference is surprising as diabetes-related complications have predominantly a vascular aetiology, with South Asian patients at higher risk of cardiovascular disease compared to White Caucasians with type 2 diabetes [20]. However, the previous reports that identified this difference either conducted a very limited assessment of DPN [17, 20] or utilized a patient population with a low prevalence of diabetes complications and could not detect a difference in DPN prevalence using routine clinical examination techniques [18]. Critically, the underlying factors that could explain such differences in DPN prevalence have not been fully explored. One previous report, based on a primary care population in the UK, suggested that differences in height and transcutaneous partial pressure of oxygen (TCpO2) were responsible for the lower prevalence of DPN in South Asians [18]. This report, however, did not adjust for a wide range of possible confounders such as obesity, alcohol intake, and triglycerides (amongst others), all of which are well established risk factors for neuropathy.

In contrast to DPN, the impact of ethnicity on CAN in patients with type 2 diabetes has received little attention, with the exception of a single report that compared the *E*/*I* ratios between South Asians and White Caucasians [18]; hence there is need for further studies utilizing more sensitive and detailed assessments of CAN.

The primary aim of this study was therefore to explore the possible metabolic, demographic, and microvascular differences that might explain the lower prevalence of DPN in South Asians with type 2 diabetes compared to White Caucasians. Secondary aims included comparing the prevalence of clinically detectable DPN and that of cardiac autonomic neuropathy (CAN) in South Asians and White Caucasians with type 2 diabetes receiving hospital-based diabetes care.

## 2. Materials and Methods

This is a secondary analysis of an ongoing project with the aim of exploring the mechanisms contributing to the development of microvascular complications in patients with type 2 diabetes [21–24]. We have conducted a cross-sectional study of casually selected South Asian or White Caucasian adults with type 2 diabetes recruited from the clinic of a hospital-based diabetes centre in the UK. Subjects excluded were those under the age of 18, not of either South Asian or White Caucasian ethnicity, or known to have neuropathy for other reasons apart from diabetes (<1%).

Patients were recruited casually from the outpatient diabetes departments of two different hospitals in the UK. The majority of participants (86%) were recruited from the Heart of England NHS Foundation Trust in Birmingham, UK, which has a large South Asian population with type 2 diabetes (approximately 40% of the total clinic). These factors suggest that our sample is representative and the two ethnicities are comparable. Patients who originated from India, Pakistan, Sri Lanka, or Bangladesh were considered to be of South Asian origin. Ethnicity was determined using the UK decennial census by the study participants. The project was approved by the Warwickshire Research Ethics Committee (REC number 08/H1211/145). Patients were consented prior to participation in the study.

DPN was assessed using the Michigan Neuropathy Screening Instrument (MNSI) which is a validated, 2-component tool designed to facilitate the early diagnosis of DPN [4, 25–29]. The questionnaire component (MNSIq) comprises 15 questions seeking to characterize sensory disturbance but also peripheral vascular disease and general asthenia [25]. The examination component (MNSIe) comprises a limited foot inspection to identify deformity, skin abnormalities, and ulceration, coupled with an assessment of the vibratory perception and ankle tendon reflexes [25]. For the purpose of this study, DPN was diagnosed if the MNSIe score was >2 and/or MNSIq was ≥7 [27]. We have also used perception to a 10 g monofilament (applied to 10 positions, the tip of each toe, under 3 metatarsal heads, the plantar surface of the foot, and the dorsal space between the first and second toe) as a test for foot insensitivity; an abnormal monofilament test was defined as < 8 correct responses [30]. The relationship of ethnicity to neuropathic symptoms was also assessed using the MNSIq.

CAN was assessed using heart rate variability (HRV) and was analyzed using the continuous wavelet transform methods to generate numerical and graphical data using the ANX-3.0 software, ANSAR Inc., Philadelphia, PA. The R-R intervals were recorded and the HRV was plotted in the frequency domain to separate the high-frequency (Rfa, 0.15 to 0.4 Hz) from the low-frequency (Lfa, 0.04 to 0.15 Hz) components by spectral analysis. HRV and BP were recorded with the patient in sitting position during resting, deep breathing, Valsalva maneuver, and standing position [31]. Data recorded included the *E*/*I* (expiratory/inspiratory) ratio, Valsalva ratio, 30 : 15 ratio, frequency domain analysis with respiratory adjustment (adjusted low frequency (Lfa), adjusted respiratory frequency (Rfa), and Lfa/Rfa), and time domain analysis including standard deviation of beat-to-beat intervals (sdNN), root mean square of successive differences (rmsSD), and the proportion of NN50 (pNN50). For the purpose of this study, a diagnosis of CAN was made when 2 or more of the following tests were abnormal: *E*/*I* ratio, Valsalva ratio, 30 : 15 ratio, and postural drop in BP (drop of 20 mmHg in systolic or 10 mmHg in diastolic BP) [2]. Age-related normal values for *E*/*I*, Valsalva, and 30 : 15 ratios were defined as previously reported [32].

Microvascular and endothelial assessment were performed on a casually chosen subset of patients (*n* = 71) using Laser speckle contrast imaging (Moor Instruments Ltd., Devon, UK) which has been shown to be reproducible [33]. All patients were approached to have the vascular/endothelial assessment and the test was performed on all those who agreed. The South Asians and White Caucasians in this subset had similar characteristics to those in the total study population reported in Table 1. Microvascular blood flow (measures in arbitrary perfusion units (APU)) was assessed at the left mid-thigh level. This site was chosen to minimise the impact of any peripheral vascular disease in the lower limbs. Measurements were taken with the patient in a semirecumbent position in a room temperature of 22-23°C following 15 minutes of patients acclimatisation [34]. Imaging was performed over 20 minutes, and all measurements were taken simultaneously. Blood flow was measured at baseline (for 20 minutes) and following maximal dilatation following heating to 44°C. Endothelial function was measured following the iontophoresis of 1% acetylcholine (Ach) and 2% sodium nitroprusside (SNP) (5 pulses over 5 minutes). Data are presented as absolute values, conductance (measured as flux divided by mean arterial pressure in order to account for differences in BP) and as percentage of maximal dilatation flow as recommended by previous reports [34]. Heating response represented maximum dilatation [34].

Data analysis was performed using SPSS 15.0 software (SPSS Inc., Chicago, USA). Data are presented as mean ± 1SD or median (IQR) depending on data distribution. Normality testing was performed using histograms and the Shapiro-Wilk test. Independent continuous variables were compared using the independent *t*-test or the Mann–Whitney test. Categorical variables were compared using the Chi square test. The Bonferroni correction was applied to define statistical significance when the components of the MNSIe and MNSIq were compared between the ethnicities. In order to identify independent predictors of DPN and CAN (separately), logistic regression models were applied. Variables included in logistic regression models were tested for multicollinearity using the tolerance test and the variance inflation factor; no evidence of multicollinearity was found in the logistic regression models used in this paper. Adiposity measures (BMI, waist, and neck circumferences) were included in the regression models separately due to the high correlation between these variables. A *p* value <0.05 was considered significant throughout the manuscript except when stated otherwise.

## 3. Results

Patients (*n* = 266) were included in this analysis, 47.4% of whom were South Asian. South Asians were shorter and younger but had similar duration of known diabetes. South Asians also had lower adiposity measurements, BP, TSH levels, smoking, and alcohol intake (Table 1). The prescription of antihypertensive and GLP-1 analogue treatment was also lower in South Asians (Table 1). The prevalence of other microvascular complications and past medical history of coronary artery disease was similar between ethnicities, while South Asians had lower prevalence of peripheral vascular disease (PVD) (Table 1) which is consistent with other previous reports [17].

DPN was more common in White Caucasians compared to South Asians (54.3% versus 38.1%, *p* = 0.008). Foot insensitivity, as assessed by abnormal monofilament perception, was also more common in White Caucasians (43.9% versus 23.8%, *p* = 0.001) (Table 2). Analysis of patient symptom scores demonstrated that symptoms consistent with sensory deficit were not different between ethnic groups whereas pain/discomfort symptoms related to were nonsignificantly more common in South Asians (Table 2). White Caucasian patients had more abnormalities on all aspects of neuropathy examination and, consistent with our findings with the monofilament, reported more open sores on the foot (Table 2).

To determine the potential influence of demographic and metabolic differences between ethnicities on the prevalence of DPN, logistic regression models with increasing complexity were used (Table 3). The association between ethnicity and DPN remained significant, despite adjusting for a wide range of possible confounders. This association was attenuated by age and a history of PVD and was abolished after adjusting for height and adiposity measures (with the exception of waist/hip ratio) (Table 3), suggesting that ethnic differences in DPN prevalence can be mainly explained by the differences in height and adiposity between the ethnic groups. All adiposity measures were independently associated with DPN despite adjusting for all possible confounders listed in Table 3 (except height and BMI which were not used in the same model) [BMI: odds ratio (OR) 1.076 (95% CI 1.026 to 1.130), *p* = 0.003; waist circumference: OR 1.040 (95% CI 1.016 to 1.064), *p* = 0.001; neck circumference: OR 1.143 (95% CI 1.045 to 1.250), *p* = 0.003]. Other independent associations included previously reported measures such as age, diabetes duration, and insulin treatment [13, 14, 35].

Unlike clinically apparent DPN, we found no difference in prevalence of abnormalities of standardized cardiovascular autonomic function tests between ethnicities (40.9% versus 40.9%, *p* = 0.995). Frequency and time domain analysis, however, revealed more extensive deficits in the White Caucasian compared to the South Asian cohort (Table 4). However, these differences were not significant after adjusting for possible confounders (such as age). Diabetes duration [OR 1.084 (95% CI 1.028 to 1.144), *p* = 0.003] and the use of calcium channel blockers [OR 0.292 (95% CI 0.123 to 0.694), *p* = 0.005] were found to be independently associated with CAN after adjusting the data, but not ethnicity [OR 1.077 (95% CI 0.473 to 2.452), *p* = 0.860].

Microvascular and endothelial assessment demonstrated significant differences between the two ethnicities. South Asians had similar baseline skin flux and conductance and markedly lower maximal dilatation following heating despite a similar SNP response but had preserved endothelial-dependant vasodilatation (Table 5). Indeed, endothelial-dependent vasodilatation significantly exceeded that measured in the White Caucasian population when expressed as the ratio of Ach-induced to heating-induced flux [34] (Table 5).

## 4. Discussion

We have compared the prevalence of DPN and that of CAN between South Asians and White Caucasians with type 2 diabetes from a hospital-based care setting. We then tried to determine whether specific metabolic and/or demographic factors could explain observed differences in the prevalence of clinically detectable DPN and CAN between these groups. Our study clearly demonstrates that DPN and foot insensitivity are less common in South Asian patients and implicates height and adiposity as potential factors underlying this difference. Furthermore, we found that total body (BMI), visceral (waist circumference), and upper body (neck circumference) fat were independently associated with DPN after adjusting for a range of confounders.

Our diabetes centre serves an area that has a high proportion of South Asians, although the majority of the general population are White Caucasians (a reflection of the much higher prevalence of type 2 diabetes in South Asians in the UK and elsewhere). Both ethnicities live in the same geographical area, have similar deprivation scores, and are referred to our clinic by primary care physicians using the same referral guidelines which are preagreed with our centre. Furthermore, there are no differences in clinic attendance rates between the ethnicities at our centre. Moreover, our population of South Asians and White Europeans with type 2 diabetes has similar characteristics (such as age, gender, smoking, alcohol, BMI, and HbA1c) to those described in previous reports in primary care in other localities in the UK. As our study population was hospital-based and with consequently more advanced disease, our findings are not necessarily generalizable to the general population of patients with type 2 diabetes (please see below) [18].

Our data demonstrating a lower prevalence of DPN in South Asian subjects are consistent with other reports [17–19] but extend these findings by demonstrating significant differences detectable by routine clinical examination and the novel finding that obesity (and its distribution) explains, at least in part, the ethnic differences in DPN prevalence. In addition, previous study populations exploring the relationships of DPN and ethnicity were drawn from Primary Care Practices in the UK which was reflected in the shorter duration of diabetes, the lower prevalence of microvascular, and macrovascular complications and the less frequent use of insulin and lipid lowering treatments [18, 19], while our study extends the findings to hospital-based population. Our study patients were drawn from a population receiving hospital-based diabetes care and are therefore representative of a more advanced, complicated disease, which has not previously been assessed. This difference in population characteristics may underlie the previously reported inability to detect significant differences in DPN prevalence using the Neuropathy Disability Score (NDS), instead relying upon findings from nerve electrophysiology [18]. Moreover, although not directly assessed, preservation of the microcirculation was implicated as a potential contributor to the lower risk of DPN and foot ulceration observed in South Asian patients [18]. There was, however, no adjustment performed for adiposity.

Our study confirms previous reports that foot ulceration is lower in South Asian compared to White Caucasian patients with type 2 diabetes [17]. This finding is consistent with our observation of relative preservation of 10 g monofilament perception in South Asian patients. Interestingly, the overall prevalence of neuropathic symptoms ascribable to DPN, however, was comparable across ethnicities with similar percentages reporting symptoms consistent with sensory loss but (nonsignificantly) more South Asian patients reporting neuropathic pain. It is known that South Asians have a higher prevalence of vitamin D deficiency [36], which might contribute to differences in some symptoms, particularly generalized weakness.

Our results showed that age, diabetes duration, and insulin treatment were independently associated with DPN, as has been previously reported [13, 14, 35]. The relationship between DPN and insulin is interesting, but due to the cross-sectional nature of this study, causation cannot be proven. Intensive insulin treatment by improving metabolic control has been shown to have beneficial effects on DPN [12]. Patients might have been started on insulin because of the presence of DPN and other microvascular complications in order to slow progression. On the other hand, our data do not show a relationship between DPN and other risk factors such as triglycerides [37]; this could be related to our sample size, the different characteristics of our study population, or the high proportion of patients that were on lipid lowering therapy.

South Asians have different body fat distribution to White Europeans and tend to have greater fat mass than White Europeans for the same BMI level [38–40]. In order to address this issue, we used different measures of adiposity including BMI, waist circumference, and neck circumference, all of which were greater in White Europeans compared to South Asians in our study. All of these adiposity measures removed the impact of ethnicity on DPN when added to the regression model.

Our data, demonstrating BMI, waist circumference, and neck circumference as independent predictors of DPN, is potentially of mechanistic importance. Neck circumference is associated with cardiometabolic risk factors [41] and with obstructive sleep apnoea (OSA) [42], a disease that is very common in type 2 diabetes (up to 86% prevalence) [43] and is known to be associated with endothelial dysfunction and abnormal blood flow regulation [44]. Interestingly, neck circumference has recently been identified as an independent predictor of diabetic retinopathy [45].

Maximal skin blood flow of the lower limb on heating was reduced but endothelial function preserved in South Asian compared to White Caucasians with type 2 diabetes. In contrast, there were no significant differences in the response to SNP. To our knowledge, this is the first evaluation of skin microvascular flow regulation in South Asian patients with diabetes. Heating to 44°C leads to maximal vasodilatation which corresponds to the maximal vasodilatory capacity [34]. Deficits in heating responses have been associated with increased prevalence of cardiovascular disease [46, 47] and so our findings might have relevance for the higher cardiovascular disease risk observed in South Asians with type 2 diabetes [20].

In contrast to the heating response, the response to Ach (when measured as a ratio of maximal vasodilatation) was greater in South Asians, suggesting relative preservation of endothelial-dependent vasodilatation. The response to Ach is thought to reflect both an axon reflex and prostaglandin-mediated component [34] which might, therefore, be of relevance to the lower prevalence of DPN and foot complications in these subjects. In patients* without* diabetes, heating response was not different [47] while SNP was lower [48] in South Asians compared to White Caucasians when measured in the forearm. Our data are also consistent with the finding of relative preservation of TCpO2 in South Asian subjects with diabetes [18].

Unlike DPN, there was no difference in the prevalence of abnormalities of standardized CAN tests between South Asian and White Caucasian patients, although differences in spectral analysis suggested more preserved autonomic function in South Asians. This highlights the fact that the pathogenesis of DPN and CAN may not be the same and understanding such differences will be important to develop targeted treatments. Differences in CAN prevalence between South Asian and White Caucasian patients with type 2 diabetes have been previously assessed using the *E*/*I* ratio and the postural drop in BP as markers of CAN [18]. In this previous report, there was no difference in the postural drop between ethnicities (consistent with our findings), but there was a significant difference in the change in heart rate associated with deep breathing between ethnicities. It is difficult to directly compare our results with the results from this report, as we performed a more extensive assessment of CAN and we have presented our *E*/*I* ratio data as categorical variable rather than an absolute change in heart rate.

There are some limitations of our study, particularly the differences in baseline parameters such as age, height, BP, smoking, and alcohol consumption between the ethnic groups which might have affected the associations observed in our study. However, the principal findings of our study remained significant after adjusting for a wide range of possible confounders. Matching ethnicities for some of the baseline parameters might have been preferred, but such precise matching is difficult (particularly regarding age and adiposity measures) due to the significant demographic differences that exist between our local South Asian and White Caucasian population with type 2 diabetes. The relatively small size of our sample might have obscured the relationship between CAN and ethnicity. Nonetheless, this sample size was adequate to detect a difference in the prevalence of DPN consistent with the construct that differences in the pathogenesis of peripheral and autonomic neuropathy may exist. In addition, this is a cross-sectional study and hence causation cannot be proven. Additionally, there is the possibility of self-selection bias (i.e., patients with microvascular complications preferentially agreeing to participate), which cannot be entirely excluded. However, all patients attending a general diabetes clinic were approached about willingness to enrol in the study and we are unaware of an ethnicity-mediated self-selection bias which would affect our conclusions. This is further supported by the similar prevalence of diabetic retinopathy between the two ethnicities. Finally, the MNSI is not the “gold standard” for diagnosing DPN but it has been validated against nerve conduction studies [25, 29] and has been used widely in landmark studies [4, 26, 27, 30]. We chose to use the MNSI (in concert with the 10 g monofilament) since it offers the advantage of providing robust, meaningful, clinically detectable end-points.

In summary, clinical signs consistent with DPN are reduced in South Asian compared to White Caucasian patients with type 2 diabetes receiving hospital-based diabetes care. In contrast, the overall prevalence of neuropathic symptoms ascribable to DPN was similar in these ethnic groups. Differences in height and adiposity appear to explain the ethnic differences in DPN prevalence; and obesity was an independent determent of DPN in these populations. We have also identified a novel association between neck circumference and DPN which might be of pathogenic importance as neck circumference is associated with cardiometabolic risk factors and OSA. Endothelial-dependent microvascular function, but not the response to heating, was also relatively preserved in South Asian subjects. Future studies are warranted to characterize small nerve fibre loss and dysfunction in different populations, address the possible role of OSA in the pathogenesis of DPN, and further explore differences in microvascular blood flow regulation between different ethnicities.

## Figures and Tables

**Table 1:** Summary of baseline characteristics in relation to ethnicity. Data are presented as median (IQR) or mean ± 1SD depending on data distribution. The percentages represent % of participants in the ethnic group. STR: sight threatening retinopathy defined as preproliferative or proliferative retinopathy or maculopathy or previous laser treatment. TIA: transient ischaemic attack. PVD: peripheral vascular disease.

	South Asians (*n* = 126)	White Caucasians (*n* = 140)	*p* value
Age (years)	54.9 ± 12.5	59.2 ± 10.8	0.003
Male (%)	60.3	56.4	0.521
Smoking (current or ex-smoker)	30.2	50.7	0.001
Alcohol (%)	2.4	47.9	<0.001
Diabetes duration (years)	11.0 (7.0–18.0)	10.5 (5.0–16.0)	0.153
BMI (kg/m^2^)	30.1 (26.5–33.6)	35.3 (31.8–41.4)	<0.001
Waist circumference (cm)	103.2 (96.9–113.1)	117.2 (109.2–129.4)	<0.001
Hip circumference (cm)	102.5 (97–112.25)	120.0 (108.6–131.75)	<0.001
Waist/hip ratio	1.00 ± 0.06	0.99 ± 0.11	0.276
Neck circumference (cm)	39.0 (36.9–41.8)	43.0 (39.6–47.0)	<0.001
Height (cm)	164.5 ± 8.9	167.1 ± 12.3	0.051
Neck height ratio	0.24 ± 0.02	0.26 ± 0.03	<0.001
Systolic blood pressure (mmHg)	126.0 (115.5–138.5)	130.0 (124.5–140.0)	0.008
Diastolic blood pressure (mmHg)	77.5 (70.0–84.5)	79.0 (73.6–85.9)	0.032
HbA1c (%)	8.0 (7.16–9.20)	8.0 (7.23–9.19)	0.868
Total cholesterol (mmol/L)	3.7 (3.3–4.3)	3.7 (3.3–4.4)	0.637
Triglycerides (mmol/L)	1.8 (1.1–2.4)	1.7 (1.3–2.5)	0.550
HDL (mmol/L)	1.1 (0.9–1.2)	1.1 (0.9–1.3)	0.217
TSH (mU/L)	1.6 (1.0–2.3)	1.9 (1.4–2.3)	0.024
Estimated GFR (mL/min/1.73 m^2^)	90.0 ± 25.5	83.7 ± 27.1	0.053
Oral antidiabetes agent (%)	91.3	92.9	0.632
Sulphonylureas (%)	42.1	30.7	0.054
Insulin (%)	50.0	57.9	0.199
Insulin dose (units)	80.0 (44.0–118)	67.0 (52.0–97.5)	0.649
GLP-1 analogues (%)	4.0	17.1	0.001
Lipid lowering treatment (%)	81	85	0.379
Antihypertensive therapy	74.6	84.3	0.05
Antiplatelet agents (%)	65.6	64.5	0.847
Albuminuria (%)	38.6	34.6	0.536
STR (%)	36.0	36.8	0.908
Ischaemic heart disease (%)	23.0	17.1	0.231
CABG (%)	13.5	10.0	0.375
Stroke/ TIA (%)	9.5	10.7	0.748
PVD (%)	2.4	8.6	0.029

**Table 2:** Ethnic differences in components of the MNSI and monofilament perception. Data are presented as % of abnormal test/response in the particular ethnic groups. MNSIe: the examination component of MNSI. MNSIq: the questionnaire component of MNSI. ^*∗*^These questions are not scored as part of the MNSIq. *p* < 0.01 and < 0.0033 were considered significant for differences in the components of MNSIe and MNSIq, respectively, following the Bonferroni correction. Statistical analysis in this table represents univariate analysis with no adjustments.

	South Asian (*n* = 126)	White Caucasian (*n* = 140)	*p* values
MNSIe			
Inspection	51.6	63.3	0.054
Ulcers	1.6	4.3	0.195
Ankle reflexes	35.7	56.1	0.001
Vibration	32.5	54.7	<0.001

10 g monofilament	23.8	43.9	0.001

MNSIq			
Are your legs and/or feet numb?	46.8	41.4	0.376
Do you ever have any burning pain in your legs and/or feet?	55.6	43.6	0.051
Are your feet too sensitive to touch?	24.6	26.4	0.733
Do you get muscle cramps in your legs and/or feet?^*∗*^	62.7	70.7	0.165
Do you ever have any prickling feelings in your legs or feet?	59.5	44.3	0.013
Does it hurt when the bed covers touch your skin?	14.3	11.4	0.486
When you get into the tub or shower, are you able to tell the hot water from the cold water?	7.9	10.0	0.558
Have you ever had an open sore on your foot?	11.1	25.7	0.002
Has your doctor ever told you that you have diabetic neuropathy?	20.6	27.1	0.215
Do you feel weak all over most of the time?^*∗*^	53.2	33.6	0.001
Are your symptoms worse at night?	43.7	36.4	0.230
Do your legs hurt when you walk?	59.5	60.7	0.843
Are you able to sense your feet when you walk?	8.7	17.1	0.043
Is the skin on your feet so dry that it cracks open?	38.9	46.4	0.215
Have you ever had an amputation?	2.4	8.6	0.029

**Table 3:** Assessing the impact of possible confounders on the association between ethnicity and DPN (based on MNSI) using logistic regression models with increasing complexity. The odds ratios reported are the odds for having DPN in White Caucasians to South Asians. BP: blood pressure; eGFR: estimated glomerular filtration rate; PVD: peripheral vascular disease; BMI: body mass index.

Model	Nagelkerke *R*^2^	Odds ratio	95% confidence interval	*p* value
Unadjusted: ethnicity	0.035	1.930	1.182–3.149	0.009
Model 1: ethnicity + age + gender	0.091	1.684	1.016–2.793	0.043
Model 2: ethnicity + age + gender + alcohol intake + smoking + BP + diabetes duration + HbA1c + lipids^*∗*^ + TSH + eGFR + glucose lowering treatments^$^ + antihypertensives^*£*^ + antiplatelets^@^ + lipid lowering therapy^∧^	0.224	1.987	1.069–3.693	0.030
Model 3: Model 2 + PVD	0.237	1.887	1.008–3.530	0.047
Model 4: Model 3 + height	0.244	1.766	0.932–3.348	0.081
Model 5: Model 3 + BMI	0.279	1.169	0.581–2.352	0.661
Model 6: Model 3 + waist circumference	0.290	1.077	0.532–2.180	0.837
Model 7: Model 3 + neck circumference	0.278	1.087	0.528–2.237	0.822

^*∗*^Adjustment for lipids included adjustment for total cholesterol, triglycerides, and HDL individually.

^$^Adjustment for glucose lowering treatments included adjustment for metformin, sulphonylurea, glitazones, DPP-4 inhibitors, insulin, and GLP-1 analogues individually. No other glucose lowering treatment was used in our study population.

^*£*^Adjustment for antihypertensives included adjustment for ACE inhibitors, angiotensin 2 blockers, beta blockers, alpha blockers, calcium antagonists, and diuretics individually.

^@^Antiplatelets included aspirin and clopidogrel.

^∧^Adjustment for lipid lowering therapy included adjustment for statins, ezetimibe, and fibrates individually.

**Table 4:** Differences in heart rate variability, spectral analysis, and time-domain and frequency-domain parameters between South Asians and White Caucasians with diabetes. Nonsignificant comparisons from the HRV and frequency and time domain analysis have not been included. Data are presented as median (IQR).

	South Asians (*n* = 115)	White Caucasians (*n* = 110)	*p* value
30 : 15 ratio	1.26 (1.11–1.60)	1.19 (1.08–1.37)	0.027
Baseline Lfa	0.95 (0.42–2.14)	0.60 (0.27–1.38)	0.041
Baseline Rfa	0.54 (0.21–1.19)	0.35 (0.15–0.80)	0.032
Deep breathing Lfa	0.70 (0.29–1.84)	0.44 (0.16–1.35)	0.035
Standing Lfa	1.13 (0.41–2.73)	0.69 (0.23–1.43)	0.018

**Table 5:** Assessment of microvascular blood flow and endothelial function in South Asians and White Caucasians with type 2 diabetes. Data presented as median (IQR) or ratios. Blood flux was measured in arbitrary perfusion units (APU). Conductance is the measure of dividing flux by the mean arterial pressure.

	South Asians (*n* = 33)	White Caucasians (*n* = 38)	*p* value, unadjusted
*Flux*			
Baseline	23.10 (17.30–35.15)	24.20 (15.70–35.22)	0.986
Heating	140.10 (107.30–169.05)	174.00 (143.12–207.62)	0.006
Ach	112.00 (84.80–135.50)	107.40 (73.62–142.77)	0.991
SNP	102.80 (66.70–141.10)	127.30 (81.60–172.60)	0.170

*Conductance*			
Baseline	0.27 (0.18–0.40)	0.27 (0.17–0.37)	0.653
Heating	1.58 (1.22–1.90)	1.77 (1.50–2.19)	0.029
Ach	1.26 (0.88–1.38)	1.07 (0.77–1.53)	0.612
SNP	1.12 (0.75–1.51)	1.30 (0.88–1.84)	0.273

*Flux in relation to maximum vasodilatation*			
Baseline	0.17 (0.12–0.24)	0.15 (0.10–0.21)	0.074
Ach	0.78 (0.62–0.91)	0.63 (0.43–0.76)	0.01
SNP	0.78 (0.46–1.01)	0.75 (0.51–0.94)	0.596

## Abbreviations

DN: Diabetic neuropathy
DPN: Diabetic peripheral neuropathy
DAN: Diabetic autonomic neuropathy
CAN: Cardiac autonomic neuropathy
TCpO2: Transcutaneous partial pressure of oxygen
MNSI: Michigan Neuropathy Screening Instrument
HRV: Heart rate variability
VLF: Very low frequency
LF: Low frequency
HF: High frequency
NDS: Neuropathy Disability Score
AGE: Advanced glycation end-product.
